# Association between baseline and changes in high-sensitive C-reactive protein and metabolic syndrome: a nationwide cohort study and meta-analysis

**DOI:** 10.1186/s12986-021-00632-6

**Published:** 2022-01-06

**Authors:** Qingping Xue, Xue Yang, Yuli Huang, Dongshan Zhu, Yi Wang, Ying Wen, Jian Zhao, Yanjun Liu, Chun-Xia Yang, Jay Pan, Tong Yan, Xiong-Fei Pan

**Affiliations:** 1 Department of Epidemiology and Biostatistics, School of Public Health, Chengdu Medical College, Chengdu, Sichuan China; 2 HEOA Group, Institute for Healthy Cities and West China Research Center for Rural Health Development, Sichuan University, Chengdu, Sichuan China; 3 Department of Epidemiology and Biostatistics, West China School of Public Health and West China Fourth Hospital, Sichuan University, Chengdu, Sichuan China; 4 Department of Cardiology, Shunde Hospital, Southern Medical University, Foshan, Guangdong China; 5 The George Institute for Global Health, Faculty of Medicine, University of New South Wales, Sydney, NSW Australia; 6 Center for Health Management and Policy Research, School of Public Health, Cheeloo College of Medicine, Shandong University, Jinan, Shandong 250012 China; 7 Department of Epidemiology and Biostatistics, Ministry of Education Key Laboratory of Environment and Health and State Key Laboratory of Environmental Health (Incubation), School of Public Health, Tongji Medical College, Huazhong University of Science and Technology, Wuhan, Hubei China; 8 Department of Communicable Diseases Control and Prevention, Shenzhen Center for Disease Control and Prevention, Shenzhen, Guangdong China; 9 The Ministry of Education – Shanghai Key Laboratory of Children’s Environmental Health, Xinhua Hospital, Shanghai Jiao Tong University School of Medicine, Shanghai, China; 10 Center of Gastrointestinal and Minimally Invasive Surgery, Department of General Surgery, The Third People’s Hospital of Chengdu and The Affiliated Hospital of Southwest Jiaotong University, Chengdu, Sichuan China; 11 Center for Obesity and Metabolic Health, The Third People’s Hospital of Chengdu and The Affiliated Hospital of Southwest Jiaotong University, Chengdu, Sichuan China; 12 Key Laboratory of Birth Defects and Related Diseases of Women and Children (Sichuan University), Ministry of Education, West China Second University Hospital, Sichuan University, Chengdu, Sichuan China

**Keywords:** High-density C-reactive protein, Metabolic syndrome, Chinese, Cohort study, Meta-analysis

## Abstract

**Background:**

We aimed to prospectively evaluate the associations between the baseline and changes in high-density C-reactive protein (hs-CRP) and incident metabolic syndrome (MetS) in China and update the evidence based on a meta-analysis of cohort studies in different populations.

**Methods:**

Data from the China Health and Retirement Longitudinal Study among adults aged 45 years or older were analyzed. Participants who were recruited in the study in 2011–2012 without MetS and successfully followed up to 2015–2016 were included in our final analysis. Logistic regressions were applied to examine the prospective associations of baseline and changes in hs-CRP with incident MetS and estimate corresponding odds ratios (ORs) and 95% confidence intervals (95% CIs). A meta-analysis was conducted to synthesize effect estimates from our findings and other cohort studies on this topic.

**Results:**

Among 4,116 participants, 535 developed MetS after a 4-year follow-up. Compared with the participants with hs-CRP in the lowest quartile, those with hs-CRP in the second, third, and highest quartiles had higher odds of MetS, with multivariable-adjusted ORs (95% CIs) of 1.51 (1.12, 2.06), 1.50 (1.11, 2.04), and 1.83 (1.37, 2.47). For the hs-CRP changes, ORs (95% CIs) were 3.24 (2.51, 4.02), 3.34 (2.56, 4.38), and 3.34 (2.54, 4.40) respectively. One unit (log of 1 mg/L) increase in hs-CRP was associated with 23% higher risk of MetS (OR 1.23; 95% CI 1.10, 1.38). In a meta-analysis of 6 cohort studies, the pooled relative risk for MetS was 1.63 (1.38, 1.93) for the highest versus lowest level of hs-CRP. In addition, the pooled relative risk for MetS was 1.29 (1.05, 1.59) for each unit increase of hs-CRP after log-transformation.

**Conclusions:**

Both higher baseline hs-CRP and longitudinal hs-CRP increases were associated with higher risks of incident MetS. Individuals with high hs-CRP levels may need to be closely monitored for future risk of MetS.

**Supplementary Information:**

The online version contains supplementary material available at 10.1186/s12986-021-00632-6.

## Background

Metabolic syndrome (MetS) is characterized by a constellation of interrelated metabolic risk factors, including abdominal obesity, hypertension, high blood glucose levels, and dyslipidemia [1]. It is associated with an approximate twofold increased risk for cardiovascular disease and a fivefold increased risk for incident type 2 diabetes mellitus [2]. With a rapid increase of MetS worldwide, it is imperative to identify and manage the medical condition in its early stage.

Although the understanding of pathophysiological mechanisms for MetS is evolving, some evidence suggests that inflammation is involved in the development of MetS. High-sensitivity C-reactive protein (hs-CRP), known as an acute-phase reactant produced by liver in response to the presence of the pro-inflammation cytokines [3], is positively associated with MetS in several cohort studies in the US [4], European [5] and Asian countries [6, 7]. Most extant cohort studies were conducted in non-Chinese populations, and one cohort study among 886 southern Chinese adults with a five-year follow-up found that a high hs-CRP level was related with an increased risk of incident MetS [6]. However, this study was subject to several limitations, such as a small sample size, a narrow sampling frame, and a short follow-up. In addition, most studies on the effects of hs-CRP on cardiometabolic diseases only used a baseline measure of hs-CRP without considering the dynamic changes of hs-CRP. One study from the US showed increased risks of incident diabetes, cardiovascular disease, and mortality in relation to sustained elevated hs-CRP [8], while a separate study from China found that adults with “moderate-increased” hs-CRP (moderate at baseline and increased to high concentration at follow-ups) had a higher risk of incident diabetes, compared with those with “low-stable” hs-CRP (low at baseline and maintained at low concentration at follow-ups) [9]. These findings suggest that longitudinal changes, particularly sustained elevations of hs-CRP may carry extra information for chronic inflammation compared with single hs-CRP measurements. However, no prior studies ever explored the association of longitudinal changes in hs-CRP with incident MetS. Given the high prevalence of MetS (24.5%) in Chinese adults [10], it is necessary to systematically assess the link of hs-CRP to MetS risk using national population-based longitudinal data in China.

Thus, our research aimed to use the data from the China Health and Retirement Longitudinal Study (CHARLS) to prospectively examine the associations of baseline hs-CRP and longitudinal hs-CRP changes with incident MetS among middle-aged and elderly Chinese and assess whether the associations differed in subpopulations. Since prior prospective studies on this topic from different populations contained mixed findings [11, 12], we pooled our findings with reports from previous cohort studies to corroborate our work.

## Methods

### Study population

The CHARLS was conducted in a national representative sample of adults aged 45 years or older in China. The study design, sampling procedures and survey methods of the CHARLS were reported in details elsewhere [13]. Briefly, based on a probability-proportional-to-size sampling design, a total of 17,708 participants were recruited from 150 counties of 28 provinces at baseline between June 2011 and March 2012. At enrollment, data were collected by trained staff using standardized questionnaires on demographic and socioeconomic characteristics, health conditions, health-related lifestyle and behaviors, and medical conditions. Physical measurements and blood samples were also obtained at baseline. The participants were followed up until 2015–2016, and similar procedures for data collection were repeated in the 2015–2016 follow-up. Plasma samples were aliquoted and immediately frozen at − 20 °C, and transported to the Chinese Center for Disease Control and Prevention in Beijing within 2 weeks where they were placed in a deep freezer and stored at − 80 °C until assayed at the Capital Medical University laboratory. The CHARLS was approved by the Biomedical Ethics Review Committee of Peking University (IRB00001052-11,015), and informed consent was obtained from all study participants.

Our analyses utilized prospective data from the CHARLS collected between 2011 and 2016. Among 17,708 participants in the CHARLS, 11,847 completed both questionnaires and laboratory measurements at baseline. In our work, we excluded (1) participants without information for MetS at baseline (n = 2028); (2) participants with MetS at baseline (n = 2740); (3) participants with hs-CRP ≥ 10 mg/L at baseline (n = 276) as high levels of hs-CRP could be caused by acute inflammation [14]; (4) participants who were lost to follow-up (n = 2219); (5) participants without information of MetS or hs-CRP in 2015–2016 (n = 87); and (6) participants with data missing for age (n = 177), residential area (n = 4), cigarette smoking (n = 12), alcohol drinking (n = 3), BMI (n = 33), diabetes (n = 60), hypertension (n = 19), dyslipidemia (n = 53), heart diseases (n = 12), or stroke (n = 8). A total of 4,116 who had no MetS at baseline and were followed up successfully until 2015–2016 were included in final analyses (Additional file 1: Fig. S1).

### Definition of MetS

MetS was diagnosed according to the Chinese guidelines for the management of dyslipidemia in adults [15] as having three or more of the following items: (1) waist circumference (WC) ≥ 90 cm of men or ≥ 80 cm of women; (2) fasting blood glucose (FBG) levels ≥ 110 mg/dl (6.10 mmol/L) or undergoing anti-diabetes treatment; (3) systolic blood pressure (SBP) ≥ 130 mm Hg or diastolic blood pressure (DBP) ≥ 85 mm Hg or undergoing anti-hypertensive drug treatment; (4) fasting triglycerides (TG) ≥ 150 mg/dl (1.7 mmol/L); and (5) fasting high-density lipoprotein cholesterol levels (HDL-C) < 40 mg/dl (1.0 mmol/L). Incident MetS was defined as new-onset MetS during the follow-up in 2015–2016.

### Assessment of hs-CRP

Hs-CRP was measured by the immunoturbidimetric assay at baseline and in the 2015–2016 follow-up, and its analytical range was 0.1–20.0 mg/L and between-assay coefficient of variation was < 5.7%. The same Roche kits (Roche Diagnostics, Basel, Switzerland) on a Hitachi 7180 chemistry analyzer (Hitachi, Tokyo, Japan) were used for all assays [16].

### Measurement of covariates

Covariates including year of birth, sex, education level (illiterate, primary school, middle school, and high school or above), residence (urban and rural), cigarette smoking (never, former, and current), alcohol drinking (never, former, and current), diabetes (yes and no), hypertension (yes and no), dyslipidemia (yes and no), heart disease (yes and no), and stroke (yes and no) were collected in the baseline survey. BMI was calculated as body weight in kilograms divided by height in meters squared. Prevalent diabetes was determined by fasting glucose measures (FBG ≥ 126 mg/dl or HbA1c ≥ 6.5%) or self-reported physician diagnosis [17]. Prevalent hypertension was determined by blood pressure (SBP ≥ 140 mmHg or DBP ≥ 90 mmHg) or self-reported physician diagnosis [18]. Prevalent dyslipidemia was defined as total cholesterol/HDL-C > 5 or self-reported physician diagnosis [19]. Prevalent heart disease and stroke were determined by self-reported physician diagnosis.

### Statistical analysis

Mean (standard deviation, SD) were reported for continuous variables and frequency (percentage, %) were reported for categorical variables. Analysis of variance (for continuous variables) and Chi-square (for continuous variables) were used to compare basic characteristics in participants across baseline hs-CRP quartiles. Logistic regressions were performed to estimate the odds ratios (ORs) and 95% confidence intervals (CIs) for the associations between hs-CRP and incident MetS. We adjusted for age (continuous, years), sex, education level, and residence in the first multivariable model, and additionally adjusted for cigarette smoking, alcohol drinking, BMI (continuous, kg/m^2^), hypertension, dyslipidemia, diabetes, heart disease and stroke in the second multivariable model. In the main analyses, hs-CRP levels were grouped as quartiles and the lowest quartile was set as the reference group. Elevated hs-CRP was defined as a concentration of hs-CRP higher than 3 mg/L to dichotomize hs-CRP levels [8]. In addition, we used continuous hs-CRP levels with natural log transformation as the independent variable in a separate analysis. Similar analyses were conducted to assess the associations between absolute and percent changes of hs-CRP (group 1 included participants with negative or null changes or percent changes, and group 2–4 were tertiles for the remaining participants) and MetS. The linear trend in the associations of baseline and longitudinal changes in hs-CRP with MetS was assessed by assigning the median hs-CRP or hs-CRP changes of quartiles/groups for logistic regression models. We also assessed the association of combined baseline hs-CRP and absolute hs-CRP changes with incident MetS by segmenting four groups based on whether hs-CRP was higher than 3 mg/L [8] and the median value of hs-CRP changes: low baseline/low increase (reference group), high baseline/low increase, low baseline/high increase, and high baseline/high increase.

We conducted two sensitivity analyses to confirm our results. In the first sensitive analysis, we performed multiple imputations for 381 participants with data missing for age, sex, education level, cigarette smoking, residential area, cigarette smoking, alcohol drinking, BMI, diabetes, hypertension, dyslipidemia, heart disease and stroke prior to logistic regressions. Under the assumption of missing at random, we replaced missing values by imputed ones from ten duplicate datasets which were created by imputation simulation to reduce sampling variability. Finally, we combined the effect estimates from the ten imputed datasets to obtain the pooled effect estimates and their 95% CIs for the associations of both baseline hs-CRP and hs-CRP changes with incident MetS. In the second sensitive analysis, we conducted logistic regressions by excluding participants with diabetes, heart disease and stroke.

We stratified participants by age (≤ 60 years and > 60 years), sex (male and female) and BMI (< 24 and ≥ 24 kg/m^2^ based on the Chinese criteria for overweight and obesity [20]) and added a product term of the stratifying variables and baseline hs-CRP or hs-CRP changes to the final model to examine potential effect modifications (interactions) using a likelihood ratio test.

### Meta-analysis

We conducted a meta-analysis to synthesize our findings with those from previous cohort studies that dealt with this topic. We carried out an electronic search in PubMed and Embase for cohort studies up to May 13, 2020, using a search strategy that combined MeSH terms and keywords for “metabolic syndrome”, “CRP” and “C-reactive protein” (Additional file 1: Table S1**)**. Studies were included for analyses if they met these criteria: 1) they were prospective cohort studies from general population or from patients without MetS at baseline; 2) adjusted relative risks (RRs), ORs, or hazard ratios with 95% CIs were reported for the prospective association between hs-CRP and incident MetS; and 3) the reported language was English. If the same topic was examined in multiple articles that were derived from the same cohort, only the latest published article was included for meta-analysis. The title, abstract, and full text of all identified studies were screened by two investigators (XY and QX) independently (Additional file 1: Fig. S2). Any discrepancies were resolved through discussions to reach a consensus. The Newcastle–Ottawa quality assessment scale [21] was used to assess the quality of included studies, which is primarily based on the selection of the study groups, the comparability of the groups, and the ascertainment of either the exposure or outcome of interest.

For data synthesis, basic characteristics and fully adjusted effect estimates were extracted from each study. Statistical heterogeneity was indicated by *P* < 0.01 in the Q test. Random-effects (if heterogeneity existed) or fixed-effects (if no heterogeneity existed) meta-analysis was conducted to pool RRs for the highest hs-CRP level category compared with the lowest hs-CRP level category or hs-CRP with natural log-transformation. For studies which reported separate results for men and women, we applied random-effect meta-analysis to combine the effect estimates to obtain total effect prior to final meta-analysis. In our study, we converted the ORs into RRs using the formula (RR = OR/([1 − pRef] + [pRef × OR]), where pRef is the prevalence of the outcome in the reference group [22]. Hazard ratios were regarded as approximate RRs for the meta-analysis. Sensitivity analyses were conducted to explore the influence of individual studies on the summary RRs. We used the funnel plot and Egger’s test to assess the publication bias. All statistical analyses were conducted in R (version 3.6.1) and *P*-value < 0.05 was considered to indicate statistical significance.

## Results

### Baseline characteristics

Of 4,116 participants without MetS at baseline, 53.13% were male and the mean age was 58.58 (SD, 9.03) years. The median (interquartile range) of hs-CRP was 0.81 mg/dl (0.48, 1.63) in our study, 0.76 mg/dl (0.45, 1.50) in females and 0.89 mg/dl (0.51, 1.78) in males. Compared with the lowest quartile of hs-CRP, those in the highest quartile of hs-CRP were more likely to be female, older, urban residents, smokers, and have higher BMI, WC, FBG, diastolic BP, and TG but lower HDL-C (*P* ≤ 0.02 for all; Table 1). In addition, they were more likely to have prevalent heart disease, hypertension, and dyslipidemia (*P* ≤ 0.04 for all). There were no major differences in most baseline characteristics between the included 4,116 participants and 2,687 with data missing (Additional file 1: Table S2).

**Table 1:** Baseline characteristics of study participants in the CHARLS (N = 4116)

Characteristics	Total	Quartiles of hs-CRP (mg/L)				*P* values
	(%)	Quartile 1	Quartile 2	Quartile 3	Quartile 4	
		(< 0.48)	(≥ 0.48 to < 0.81)	(≥ 0.81 to < 1.63)	(≥ 1.63)	
No. of participants	4116	1027	1019	1039	1031	
Age, mean (SD), years	58.58 (9.03)	57.09 (8.75)	58.51 (9.05)	59.01 (8.93)	59.70 (9.17)	< 0.001
Sex, male (%)	2187 (53.13)	590 (57.45)	566 (55.54)	530 (51.01)	501 (48.59)	< 0.001
Education level (%)						0.89
Illiterate	1931 (46.91)	488 (47.52)	486 (47.69)	474 (45.62)	483 (46.85)	
Primary school	965 (23.45)	239 (23.27)	233 (22.87)	248 (23.87)	245 (23.76)	
Middle school	853 (20.72)	213 (20.74)	207 (20.31)	212 (20.40)	221 (21.44)	
High school or above	367 (8.92)	87 (8.47)	93 (9.13)	105 (10.11)	82 (7.95)	
Residence, urban (%)	1237 (30.05)	281 (27.36)	294 (28.85)	316 (30.41)	346 (33.56)	0.02
Cigarette smoking (%)						< 0.001
Never smoker	2510 (60.98)	670 (65.24)	653 (64.08)	614 (59.10)	573 (55.58)	
Former smoker	305 (7.41)	55 (5.36)	69 (6.77)	78 (7.51)	103 (9.99)	
Current smoker	1301 (31.61)	302 (29.41)	297 (29.15)	347 (33.40)	355 (34.43)	
Alcohol drinking (%)						0.09
Never drinker	2397 (58.24)	622 (60.56)	595 (58.39)	592 (56.98)	588 (57.03)	
Former drinker	308 (7.48)	61 (5.94)	66 (6.48)	91 (8.76)	90 (8.73)	
Current drinker	1411 (34.28)	344 (33.50)	358 (35.13)	356 (34.26)	353 (34.24)	
BMI, mean (SD), kg/m^2^	22.66 (3.39)	21.97 (3.00)	22.66 (3.30)	22.86 (3.37)	23.13 (3.76)	< 0.001
WC, mean (SD), cm	81.35 (11.65)	79.40 (10.74)	80.95 (11.93)	82.22 (10.68)	82.80 (12.84)	< 0.001
FBG, mean (SD), mg/dl	102.66 (23.79)	100.77 (20.67)	103.50 (22.93)	102.88 (22.87)	103.48 (28.00)	0.02
Diastolic BP, mean (SD), mmHg	73.75 (11.69)	72.71 (11.41)	73.56 (11.91)	74.01 (11.59)	74.73 (11.76)	0.01
Systolic BP, mean (SD), mmHg	128.96 (51.54)	126.87 (48.62)	127.46 (43.44)	130.91 (64.44)	130.54 (46.83)	0.17
TG, mean (SD), mg/dl	101.93 (50.12)	96.76 (48.94)	100.86 (46.83)	106.56 (52.65)	103.45 (51.35)	< 0.001
HDL-C, mean (SD), mg/dl	55.46 (14.25)	58.30 (15.32)	56.06 (14.13)	53.97 (13.48)	53.53 (13.53)	< 0.001
Hypertension (%)	1,266 (30.76)	270 (26.29)	311 (30.52)	324 (31.18)	361 (35.01)	< 0.001
Dyslipidemia (%)	590 (14.33)	124 (12.07)	120 (11.78)	176 (16.94)	170 (16.49)	< 0.001
Diabetes (%)	367 (8.92)	78 (7.59)	96 (9.42)	97 (9.34)	96 (9.31)	0.40
Heart disease (%)	398 (9.67)	87 (8.47)	90 (8.83)	98 (9.43)	123 (11.93)	0.04
Stroke (%)	62 (1.51)	17 (1.66)	9 (0.88)	20 (1.92)	16 (1.55)	0.26

BMI, body mass index; BP, blood pressure; CHARLS, China Health and Retirement Longitudinal Study; FBG, fasting blood glucose; HDL-C, high-density lipoprotein cholesterol; hs-CRP, high-sensitive C-reactive protein; SD, standard deviation; TG, triglycerides; WC, waist circumference

### Association of baseline hs-CRP and hs-CRP changes with incident MetS

During a four-year follow-up, 535 out of 4,116 (13.00%) participants developed incident MetS. Compared with participants in the lowest quartile of hs-CRP, participants with hs-CRP level in the second, third and fourth quartiles were 1.51 (1.12, 2.06), 1.50 (1.11, 2.04) and 1.83 (1.37, 2.47; Table 2) times as likely to have MetS after adjustment for potential confounders. There was a linear trend across the four quartiles of hs-CRP (*P* for trend < 0.001; Table 2). Using hs-CRP as a continuous variable with natural log transformation also showed a positive association between hs-CRP levels and incident MetS (multivariable-adjusted OR, 1.23; 95% CI, 1.10, 1.38; Table 2). Participants with elevated hs-CRP (3 mg/L or higher) showed a 50% (1.50; 1.14, 1.94; Table 2) higher odds of having MetS compared with those with lower hs-CRP after adjustment for sociodemographic factors, but this association became non-significant after additional adjustment for lifestyle and behavior factors and other related diseases. There were no heterogeneities across age, sex and BMI subgroups (*P for interaction* ≥ 0.41; Table 3). In addition, multiple imputations for missing data did not show appreciable changes in effect estimates across four quartiles (Additional file 1: Table S3). Furthermore, the effect estimates across four quartiles remained similar after excluding participants with diabetes, heart disease and stroke (Additional file 1: Table S4).

**Table 2:** Association of baseline hs-CRP and longitudinal hs-CRP changes with incident MetS (N = 4,116)

Models	Cases / total (%)	Model 1	Model 2	Model 3
		OR (95% CI)	OR (95% CI)	OR (95% CI)
Hs-CRP quartiles at baseline				
Quartile 1 (< 0.48)	80/1,027 (13.85)	1.00 (Ref.)	1.00 (Ref.)	1.00 (Ref.)
Quartile 2 (≥ 0.48 to < 0.81)	130/1,019 (12.43)	1.73 (1.29, 2.33)	1.73 (1.29, 2.33)	1.51 (1.12, 2.06)
Quartile 3 (≥ 0.81 to < 1.63)	144/1,039 (12.35)	1.90 (1.43, 2.55)	1.91 (1.44, 2.56)	1.50 (1.11, 2.04)
Quartile 4 (≥ 1.63)	181/1,031 (13.32)	2.52 (1.91, 3.35)	2.53 (1.92, 3.37)	1.83 (1.37, 2.47)
*P* for trend^a^	535/4,116 (13.00)	< 0.001	< 0.001	< 0.001
Log (hs-CRP) level				
Each 1 mg/L increase	535/4,116 (13.00)	1.39 (1.26, 1.55)	1.40 (1.27, 1.56)	1.23 (1.10, 1.38)
levated hs-CRP ^b^				
No	370/3,668 (10.09)	1.00 (Ref.)	1.00 (Ref.)	1.00 (Ref.)
Yes	78/448 (17.41)	1.48 (1.13, 1.92)	1.50 (1.14, 1.94)	1.27 (0.95, 1.67)
Groups of absolute changes in hs-CRP during the follow–up^c^				
Group1 (≤ 0)	140/1,479 (9.47)	1.00 (Ref.)	1.00 (Ref.)	1.00 (Ref.)
Group 2 (> 0 to ≤ 0.41)	77/873 (8.82)	1.33 (0.96, 1.82)	2.44 (1.84, 3.24)	3.24 (2.51, 4.02)
Group 3 (> 0.41 to ≤ 1.21)	133/868 (15.32)	1.30 (0.93, 1.80)	2.41 (1.80, 3.24)	3.34 (2.56, 4.38)
Group 4 (> 1.21)	185/896 (20.65)	1.31 (0.94, 1.82)	2.51 (1.87, 3.39)	3.34 (2.54, 4.40)
*P* for trend^a^	535/4,116 (13.00)	< 0.001	< 0.001	< 0.001
Groups of percent changes in hs-CRP during the follow–up^d^				
Group 1 (≤ 0)	140/1,479 (9.47)	1.00 (Ref.)	1.00 (Ref.)	1.00 (Ref.)
Group 2 (> 0 to ≤ 60%)	113/870 (12.99)	1.98 (1.49, 2.64)	2.26 (1.69, 3.01)	3.29 (2.50, 4.37)
Group 3 (> 60% to ≤ 181%)	120/871 (13.78)	1.87 (1.39, 2.52)	2.22 (1.65, 3.00)	3.71 (2.78, 4.99)
Group 4 (> 181%)	162/996 (16.27)	1.91 (1.41, 2.58)	2.24 (1.65, 3.03)	3.76 (2.80, 5.09)
*P* for trend^a^	535/4,116 (13.00)	< 0.001	< 0.001	< 0.001

Model 1: Non-adjusted

Model 2: Adjusted for age (continuous, years), sex (male and female), education level (illiterate, primary school, middle school, and high school or above), and residence (urban and rural)

Model 3: Adjusted for the variables in Model 2 and cigarette smoking (never, former, and current), alcohol drinking (never, former, and current), BMI (continuous, kg/m^2^), hypertension (yes and no), dyslipidemia (yes and no), diabetes (yes and no), heart disease (yes and no) and stroke (yes and no)

Baseline hs-CRP (continuous, mg/L) was additionally adjusted for as a covariate in the analysis of quartiles of absolute changes (or percent changes) in SUA during the follow–up

CI, confidence interval; hs-CRP, high-sensitive C-reactive protein; MetS, metabolic syndrome; OR, odds ratio

^a^*P* values for trend were estimated by modelling serum hs-CRP using the median for each category

^b^Elevated hs-CRP was categorized into two groups: yes (higher than 3 mg/L) and no (lower than 3 mg/L)

^c^Participants were categorized into four groups: Group 1 included participants with negative or null changes, and Group 2–4 were tertiles for the remaining participants

^d^Participants were categorized into four groups: Group 1 included participants with negative or null percent changes, and Group 2–4 were tertiles for the remaining participants

**Table 3:** Association of baseline hs-CRP and longitudinal hs-CRP changes with incident MetS among age, sex and BMI subgroups (N = 4,116)

Subgroups	Quartiles of hs-CRP at baseline				*P* for interaction^a^	Groups of absolute changes in hs-CRP during the follow–up				*P* for interaction^a^
	Quartile 1	Quartile 2	Quartile 3	Quartile 4		Group 1	Group 2	Group 3	Group 4	
Age					0.41					0.66
45–60 years										
Case/total	50/692	83/630	83/597	103/560		76/862	52/545	81/543	110/529	
OR (95% CI)^b^	1.00 (Ref.)	1.70 (1.16, 2.53)	1.64 (1.11, 2.44)	2.03 (1.39, 3.00)		1.00 (Ref.)	1.62 (1.05, 2.49)	2.65 (1.78, 3.95)	3.63 (2.52, 5.27)	
> 60 years										
Case/total	30/335	47/389	61/442	78/471		64/617	25/328	52/325	75/367	
OR (95% CI)^b^	1.00 (Ref.)	1.26 (0.77, 2.1)	1.29 (0.80, 2.11)	1.57 (0.99, 2.54)		1.00 (Ref.)	0.97 (0.56, 1.66)	2.27 (1.43, 3.6)	2.92 (1.93, 4.47)	
Sex					0.66					0.68
Male										
Case/total	34/437	54/453	57/509	78/530		59/727	26/398	55/366	83/438	
OR (95% CI)^b^	1.00 (Ref.)	1.50 (0.94, 2.44)	1.25 (0.78, 2.03)	1.80 (1.15, 2.86)		1.00 (Ref.)	0.94 (0.54, 1.61)	2.52 (1.59, 4.01)	3.64 (2.42, 5.54)	
Female										
Case/total	46/590	76/566	87/530	103/501		81/752	51/475	78/502	102/458	
OR (95% CI)^b^	1.00 (Ref.)	1.55 (1.04, 2.33)	1.70 (1.15, 2.55)	1.90 (1.28, 2.84)		1.00 (Ref.)	1.70 (1.10, 2.63)	2.60 (1.75, 3.90)	3.3 (2.28, 4.83)	
BMI					0.69					0.40
BMI < 24.0 kg/m^2^										
Case/total	44/817	56/722	56/705	73/655		53/1,035	31/638	62/608	83/618	
OR (95% CI)^b^	1.00 (Ref.)	1.47 (0.97, 2.24)	1.38 (0.91, 2.11)	2.05 (1.38, 3.07)		1.00 (Ref.)	1.46 (0.88, 2.42)	3.23 (2.09, 5.05)	4.08 (2.73, 6.18)	
BMI ≥ 24.0 kg/m^2^										
Case/total	36/210	74/297	88/334	108/376		87/444	46/235	71/260	102/278	
OR (95% CI)^b^	1.00 (Ref.)	1.61 (1.02, 2.57)	1.64 (1.05, 2.60)	1.79 (1.16, 2.80)		1.00 (Ref.)	1.3 (0.83, 2.02)	2.09 (1.39, 3.15)	2.85 (1.96, 4.17)	

BMI, body mass index; CI, confidence interval; hs-CRP, high-sensetive C reactive protein; MetS, metabolic syndrome; OR, odds ratio

^a^*P* values for interaction were estimated using the likelihood ratio test for the product term of the stratifying variables and hs-CRP groups to the main model

^b^All models were adjusted for age (continuous, years), sex (male and female), education level (illiterate, primary school, middle school, and high school or above), and residence (urban and rural), cigarette smoking (never, former, and current), alcohol drinking (never, former, and current), BMI (continuous, kg/m^2^), hypertension (yes and no), dyslipidemia (yes and no), diabetes (yes and no), heart disease (yes and no) and stroke (yes and no)

Compared with participants in the lowest group of absolute hs-CRP changes, those with absolute hs-CRP changes in the second, third and fourth groups were 3.24 (2.51, 4.02), 3.34 (2.56, 4.38), and 3.34 (2.54, 4.40) times as likely to develop MetS after adjustment for potential confounders (Table 2). Similarly, compared with participants in the lowest group of percent hs-CRP changes, those with percent hs-CRP changes in the second, third and fourth groups were 3.29 (2.50, 4.37), 3.71 (2.78, 4.99), and 3.76 (2.80, 5.09) times as likely to have MetS. Linear trends were noted in these two sets of analyses (*P* for trend < 0.001). There were no heterogeneities across age, sex and BMI subgroups (*P for interaction* ≥ 0.40; Table 3). Additionally, multiple imputations for missing data did not show appreciable changes in effect estimates across four groups of hs-CRP changes (Additional file 1: Table S3). The effect estimates remained similar after excluding participants with diabetes, heart disease and stroke (Additional file 1: Table S4).

Compared with participants with low baseline/low increase of hs-CRP, those with high baseline/low increase, low baseline/high increase and high baseline/high increase were 2.17 (1.52, 3.07), 2.55 (2.04, 3.21) and 2.68 (1.40, 4.90) times as likely to have MetS (*P* for trend < 0.001, Additional file 1: Fig. S3).

### Association of baseline hs-CRP and hs-CRP changes with MetS components

Participants in the highest quartile of hs-CRP had a higher likelihood of elevated WC (multivariable-adjusted OR, 1.46; 95% CI, 1.10, 1.93), elevated TG (1.32; 1.06, 1.66) and elevated FBG (1.58; 1.18, 2.12; Table 4), compared with those in the lowest quartile of hs-CRP. Participants in the group of highest hs-CRP changes had a higher likelihood of elevated WC (multivariable-adjusted OR, 1.72; 95% CI 1.32, 2.24), elevated TG (3.08; 2.50, 3.80), elevated HDL-C (3.50; 2.57, 4.79), and elevated FBG (1.60; 1.25, 2.04; Table 4), compared with participants in the group of lowest hs-CRP changes. Additionally, there were no heterogeneities across age, sex and BMI subgroups for the association between hs-CRP and MetS components (*P for interaction* ≥ 0.19; Additional file 1: Table S5). There were generally no heterogeneities across age, sex, and BMI subgroups for the association between hs-CRP changes and MetS components (*P for interaction* ≥ 0.09), except for sex (*P for interaction* = 0.04) and BMI (*P for interaction* < 0.001) subgroups for elevated WC, age subgroups (*P for interaction* = 0.01) for elevated TG, and age subgroups (*P for interaction* = 0.02; Additional file 1: Table S6) for elevated BP.

**Table 4:** Association of baseline hs-CRP and longitudinal hs-CRP changes with components of MetS

Components	Quartiles of hs-CRP at baseline				Per 1 log mg/L increase	*P* for trend^a^	Groups of absolute changes in hs-CRP during the follow–up				*P* for trend^a^
	Quartile 1	Quartile 2	Quartile 3	Quartile 4			Group 1	Group 2	Group 3	Group 4	
Elevated WC											
Case/total	127/820	158/817	175/828	195/823	655/3,288		190/1,184	122/697	179/692	164/715	
OR (95% CI)^b^	1.00 (Ref.)	1.14 (0.86, 1.50)	1.18 (0.89, 1.56)	1.46 (1.10, 1.93)	1.17 (1.04, 1.30)	0.01	1.00 (Ref.)	1.12 (0.85, 1.47)	1.85 (1.43, 2.39)	1.72 (1.32, 2.24)	< 0.001
Elevated TG											
Case/total	181/1,020	211/1,061	233/1,064	259/1,053	884/4,198		209/1,539	137/883	248/876	290/900	
OR (95% CI)^b^	1.00 (Ref.)	1.11 (0.88, 1.39)	1.19 (0.95, 1.49)	1.32 (1.06, 1.66)	1.13 (1.04, 1.24)	0.02	1.00 (Ref.)	1.2 (0.94, 1.52)	2.56 (2.07, 3.17)	3.08 (2.50, 3.80)	< 0.001
Reduced HDL-C											
Case/total	67/1,098	86/1,096	79/1,099	78/1,101	310/4,394		67/1,556	53/939	59/935	131/964	
OR (95% CI)^b^	1.00 (Ref.)	1.19 (0.85, 1.67)	1 (0.71, 1.41)	0.91 (0.64, 1.30)	0.96 (0.84, 1.11)	0.27	1.00 (Ref.)	1.39 (0.96, 2.02)	1.56 (1.08, 2.25)	3.50 (2.57, 4.79)	< 0.001
Elevated BP											
Case/total	164/701	194/699	189/700	204/703	751/2,803		249/972	164/607	159/601	179/623	
OR (95% CI)^b^	1.00 (Ref.)	1.10 (0.86, 1.42)	0.98 (0.76, 1.27)	1.05 (0.82, 1.36)	1.04 (0.94, 1.15)	0.91	1.00 (Ref.)	1.11 (0.87, 1.40)	1.1 (0.86, 1.40)	1.14 (0.90, 1.45)	0.29
Elevated FBG											
Case/total	86/1,003	110/993	138/1,016	158/1,005	493/4,027		161/1,414	64/860	109/858	158/985	
OR (95% CI)^b^	1.00 (Ref.)	1.20 (0.89, 1.63)	1.42 (1.06, 1.90)	1.58 (1.18, 2.12)	1.22 (1.09, 1.37)	0.01	1.00 (Ref.)	0.65 (0.48, 0.88)	1.16 (0.89, 1.51)	1.60 (1.25, 2.04)	< 0.001

BMI, body mass index; BP, blood pressure; FBG, fasting blood glucose; HDL-C, high-density lipoprotein cholesterol; hs-CRP, high-sensitive C-reactive protein; MetS, metabolic syndrome; TG, triglycerides; WC, waist circumference

^a^*P* values for trend were estimated by modelling the serum high-sensitive C-reactive protein using the median for each category

^b^All models were adjusted for age (continuous, years), sex (male and female), education level (illiterate, primary school, middle school, and high school or above), and residence (urban and rural), cigarette smoking (never, former, and current), alcohol drinking (never, former, and current), BMI (continuous, kg/m^2^), hypertension (yes and no), dyslipidemia (yes and no), diabetes (yes and no), heart disease (yes and no) and stroke (yes and no)

### Meta-analysis for the association between CRP and MetS

We identified 9,902 records, including 3,980 from PubMed and 5,922 from Embase. We excluded 2,514 duplicate records, and 7,324 records based on title and abstract. Of the 64 records with full texts, we excluded 49 with non-cohort studies, 5 with non-original research (1 review, 2 conference abstracts, and 2 letters to editor), and 1 record without reporting the effect estimates. Thus a total of 10 studies (including the current one) were eligible for our meta-analysis (Additional file 1: Fig. S2).

The 10 studies comprised a total of 24,552 participants and 4,209 incident MetS cases [4–7, 11, 12, 23–25], of which five studies treated the CRP level as categorical variable, and four studies treated the CRP level as continuous variable with log transformation, and our current study in Chinese treated the CRP as both categorical and continuous variables (Additional file 1: Table 7). The Newcastle-Ottawa score for included studies ranged from 5 to 8 (out of 8), with 5 studies having moderate quality (6–7 points) and 3 studies having high quality (8 points) (Additional file 1: Table S8).

**Fig. 1:**
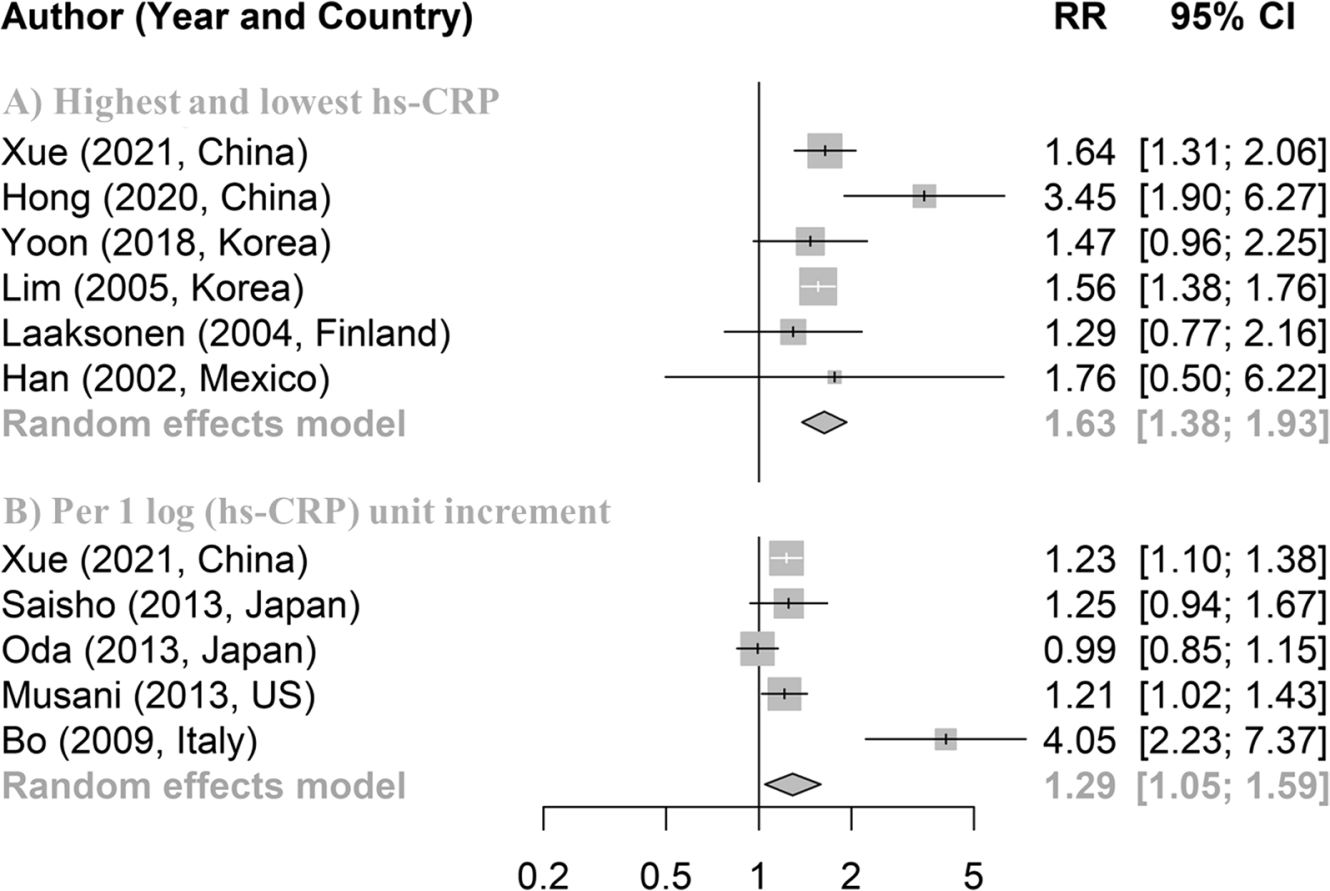
Meta-analysis for the association between hs-CRP and incident metabolic syndrome. **A**) Pooled relative risk of incident metabolic syndrome compared the highest and lowest serum hs-CRP; and **B**) Pooled relative risk of incident metabolic syndrome with log-transformed serum hs-CRP. CI, confidence interval; RR, relative rask; hs-CRP, high-sensitive C-reactive protein

The pooled RR (95% CI) for incident MetS comparing individuals in the highest versus lowest groups was 1.63 (95% CI 1.38, 1.93; *I*^2^ = 32.4%, *P* = 0.01; Fig. 1). The pooled RR (95% CI) for incident MetS for each 1 SD increase in log CRP was 1.29 (95% CI 1.05, 1.59; *I*^2^ = 82.0%, *P* < 0.001; Fig. 1). The sensitivity analyses with exclusions of a study each time further confirmed the robustness of the results in our meta-analyses (Additional file 1: Fig. S4). There was no evidence of publication bias, as *P* values for Egger’s test were 0.51 and 0.22 for two meta-analyses (Additional file 1: Fig. S5).

## Discussion

In our nationwide population-based study, we found associations of higher baseline hs-CRP levels and hs-CRP increases with elevated risks of MetS among middle-aged and elderly Chinese population during a 4-year follow-up. Of participant groups defined by baseline and changes of hs-CRP, the group with high baseline/high increase had the highest risk of incident MetS. No heterogeneities were found in subgroups stratified by age, sex and BMI. In addition, our results were further confirmed by the meta-analysis of 10 cohort studies.

In our study, participants in the highest quartile of hs-CRP had 83% increased odds of having MetS compared with those in the lowest quartile, and there was a linear trend across quartiles. Our findings were generally consistent with those from another recent study that reported a positive association between hs-CRP and MetS among 886 Chinese adults (average age, 52.0 years) during a 5-year follow-up [6]. However, this study had a relatively small sample size and was limited to residents in a small town in China. Using a nationwide population-based design, we were able to improve the robustness and generalizability of these findings in China. Similarly, one study in 9773 Korean adults aged 40–65 years [7] showed that high hs-CRP was an independent predictor for incident MetS in Asia. Consistently, two smaller studies from US (1,215 participants) [4] and Italy (201 participants) [5] also reported positive associations between hs-CRP and incident MetS. While the magnitude of association was equivalent between men and women in our study (OR, 1.80 vs 1.90), sex heterogeneity was reported before. Two studies in Chinese adults [6] and Mexicans [23] reported a positive association in women but not men in subgroup analyses. Although higher visceral fat levels [26] and sex hormone estrogen [27] in women could potentially contribute to MetS risk, the sex heterogeneity should be further examined in more large population-based cohort studies worldwide.

In addition, one novel finding of our work was the strong positive association between longitudinal increases in hs-CRP and incident MetS. Previous studies using only a single measurement of hs-CRP to represent chronic inflammation and its relationship with MetS ignored the time-varying nature of hs-CRP. Our study with two repeated measures of hs-CRP during a four-year follow-up further confirmed the positive association between hs-CRP increase and MetS risk. To our knowledge, our study has been the first to explore the relationship between hs-CRP dynamics and incident MetS in Chinese adults. Consistent with our work, a community-based cohort study among 10,160 participants in the US showed that participants with large increases or sustained elevations in hs-CRP over a 6-year period had 1.39 to 1.56 times higher risks of incident diabetes [8]. In addition, our analyses in examining the combined effects of baseline and changes of hs-CRP showed that the participants with high baseline/high increase had the highest risk of MetS among participant categories. In agreement with our finding, a separate study among 6,439 Chinese adults used latent mixture modeling to identify differential hs-CRP trajectories based on three measurements in a three-year follow-up, and demonstrated that participants in the “moderate-increased” hs-CRP group had 2.18-fold higher risk of incident diabetes, compared with those in the “low-stable” hs-CRP group [9]. Ideally, multiple repeated measurements should be collected in large cohort studies to confirm the association between hs-CRP dynamics and MetS in future.

Our meta-analysis of 10 cohort studies among 24,552 participants supported a positive association between hs-CRP and incident MetS. In particular, summary effect estimates based on both categorical and continuous variables of hs-CRP consistently confirmed the positive association. However, overall heterogeneity was observed in our meta-analysis, which could be attributable to differences in participant characteristics (such as age and sex) and research designs (such as follow-up durations and sample sizes). However, due to the small number of original studies, we did not statistically explore the subgroup heterogeneity by meta-regression. The findings from our cohort study and meta-analysis suggest that individuals with increased hs-CRP levels might need to be closely monitored for future risk of MetS in clinical practice. Of note, previous studies proposed to incorporate hs-CRP into the criteria for defining MetS, given the added value of hs-CRP for predicting incident cardiovascular diseases [28] and mortality [29]. Thus future large-scale population-based studies are still warranted to examine whether hs-CRP should be included as one component of MetS.

While the underlying mechanisms for the association between increased hs-CRP and MetS are evolving, there are certain possible explanations. First, CRP is an acute-phase reactant by the liver in response to pro-inflammation cytokines such as interleukin-6 and tumor necrosis factor-α [30]. Besides stimulating the synthesis of CRP in liver, these cytokines cause abnormalities in lipid metabolism [31] and glucometabolism [32], leading to the development of MetS. Second, CRP has an impact on insulin-stimulated phosphorylation of insulin receptor substrate 1 in vitro, suggesting that human CRP could disturb the glucose metabolism via insulin signaling pathways that regulate cell glucose transport [33]. Third, increased CRP might reduce nitric oxide bioavailability via decreasing urinary excretion of cyclic guanosine monophosphate and activating NADPH oxidase, which could aggravate hypertension and thus MetS [34, 35].

Our study has major strengths including a large sample size, a prospective population-based design, repeated measurements of hs-CRP, and a comprehensive meta-analysis. Our study has also been the first to explore the association between longitudinal changes in hs-CRP and incident MetS in China. However, some limitations should be acknowledged in our original analyses. First, a large number of participants were excluded from final analyses due to data missing and loss to the follow-up, which could lead to selection and information bias. Second, reverse causality could not be eliminated due to a short follow-up. However, our finding was corroborated by studies with longer follow-ups in other populations. Third, we failed to adjust for certain potential confounders such as physical activity, detailed medical conditions and medications due to data unavailability, so residual confounding could not be avoided. Fourth, as our study was conducted in middle-aged and elderly Chinese, our findings may not be readily generalizable to the entire Chinese population or other populations, especially the younger adults. However, our meta-analysis of multiple studies in a wider age range lends support to the generalizability of our findings.

## Conclusion

In conclusion**,** we found positive associations of baseline hs-CRP and longitudinal hs-CRP changes with incident MetS, and such positive associations were confirmed by additional meta-analyses. Our findings suggest that individuals with high hs-CRP levels may need to be closely monitored for future risk of MetS, and highlight the need to examine whether high hs-CRP levels should be incorporated into the components for MetS.

## Supplementary Information

**Additional file 1.** **Table S1:** Search strategy for PubMed and EMBASE for the meta-analysis; **Table S2:** Comparison of 4116 study participants included in final analyses and 2687 excluded due to data missing in the CHARLS; **Table S3:** Association of baseline hs-CRP and longitudinal hs-CRP changes with incident MetS after multiple imputations of missing data (N = 4497); **Table S4:** Association of baseline hs-CRP and longitudinal hs-CRP changes and incident MetS after excluding participants with diabetes, heart disease and stroke (N = 3561); **Table S5:** Association between baseline hs-CRP and components of MetS among age, sex and BMI subgroups (N = 4116); **Table S6:** Association between longitudinal hs-CRP changes and components of MetS among age, sex and BMI subgroups; **Table S7:** Basic information of studies included in the meta-analysis; **Table S8:** Quality assessment for the 10 included studies in the meta-analysis; **Figure S1:** Flowchart of inclusion and exclusion of study participants; **Figure S2:** Flowchart of literature identification for the meta-analysis; **Figure S3:** Association of combined baseline hs-CRP and longitudinal hs-CRP changes with incident MetS; **Figure S4:** Sensitivity analyses for the association between hs-CRP and incident MetS with exclusions of each study a time; **Figure S5:** Funnel plot for assessment of publication bias for the association between hs-CRP and MetS.

## Data Availability

The datasets used in the current study are available on the CHARLS official website, http://charls.pku.edu.cn/. All data, analytic methods, and study materials presented within this article are available from the corresponding authors on reasonable request.

## Abbreviations

BMI: Body mass index
BP: Blood pressure
CHARLS: China Health and Retirement Longitudinal Study
CI: Confidence interval
FBG: Fasting blood glucose
HDL-C: High-density lipoprotein cholesterol
HR: Hazard ratio
Hs-CRP: High-sensitive C-reactive protein
MetS: Metabolic syndrome
OR: Odds ratio
RR: Relative risk
SD: Standard deviation
TG: Triglycerides
WC: Waist circumference
