# Supplementary material for: Association between baseline and changes in high-sensitive C-reactive protein and metabolic syndrome: a nationwide cohort study and meta-analysis
Source: Nutr Metab (Lond). 2022 Jan 6;19:2. doi: 10.1186/s12986-021-00632-6 (PMC8734319; doi:10.1186/s12986-021-00632-6)
Supplement: Supplementary file 1 — Additional file 1. Table S1: Search strategy for PubMed and EMBASE for the meta-analysis; Table S2: Comparison of 4116 study participants included in final analyses and 2687 excluded due to data missing in the CHARLS; Table S3: Association of baseline hs-CRP and longitudinal hs-CRP changes with incident MetS after multiple imputations of missing data (N = 4497); Table S4: Association of baseline hs-CRP and longitudinal hs-CRP changes and incident MetS after excluding participants with diabetes, heart disease and stroke (N = 3561); Table S5: Association between baseline hs-CRP and components of MetS among age, sex and BMI subgroups (N = 4116); Table S6: Association between longitudinal hs-CRP changes and components of MetS among age, sex and BMI subgroups; Table S7: Basic information of studies included in the meta-analysis; Table S8: Quality assessment for the 10 included studies in the meta-analysis; Figure S1: Flowchart of inclusion and exclusion of study participants; Figure S2: Flowchart of literature identification for the meta-analysis; Figure S3: Association of combined baseline hs-CRP and longitudinal hs-CRP changes with incident MetS; Figure S4: Sensitivity analyses for the association between hs-CRP and incident MetS with exclusions of each study a time; Figure S5: Funnel plot for assessment of publication bias for the association between hs-CRP and MetS. [file 12986_2021_632_MOESM1_ESM.docx]

**Table S1. Search strategy for PubMed and Embase for the meta-analysis**

| **Search** | **Query** | **Results** |
| --- | --- | --- |
| PubMed |  |  |
| #1 | Search (metabolic syndrome) | 82,415 |
| #2 | Search (CRP) | 47,960 |
| #3 | Search (C-reactive protein) | 78,364 |
| #4 | Search (#2 OR #3) | 90,290 |
| #5 | Search (#1 AND #4) | 3,980 |
| Embase |  |  |
| #1 | Search (exp C reactive protein) | 172,742 |
| #2 | Search (exp metabolic syndrome) | 82,292 |
| #3 | Search (#1 and #2) | 5,922 |

**Table S2. Comparison of 4,116 study participants included in final analyses and 2,687 excluded due to data missing in the CHARLS**

| **characteristics** | **Total** | **Participants included** | **Participants excluded** | ***P* value** |
| --- | --- | --- | --- | --- |
| **Hs-CRP, mean (SD), mg/L** | 1.44 (1.61) | 1.39 (1.54) | 1.52 (1.72) | 0.01 |
| **Age, mean (SD), years** | 59.07 (9.74) | 58.58 (9.03) | 59.89 (10.79) | <0.001 |
| **Sex, male (%)** | 3,595 (52.85) | 2,187 (53.12) | 1,408 (52.42) | 0.58 |
| **Education level (%)** |  |  |  | <0.001 |
| Illiterate | 3,308 (48.64) | 1,931 (46.91) | 1,377 (51.28) |  |
| Primary school | 1,516 (22.29) | 965 (23.45) | 551 (20.52) |  |
| Middle school | 1,336 (19.64) | 853 (20.72) | 483 (17.99) |  |
| High school or above | 641 (9.43) | 367 (8.92) | 274 (10.20) |  |
| **Residence, urban (%)** | 2,160 (31.78) | 1,237 (30.05) | 923 (34.43) | <0.001 |
| **Cigarette smoking (%)** |  |  |  | 0.33 |
| Never smoker | 4,091 (60.40) | 25,10 (60.98) | 1,581 (59.50) |  |
| Former smoker | 524 (7.74) | 305 (7.41) | 212 (8.24) |  |
| Current smoker | 2,158 (31.86) | 1,301 (31.61) | 857 (32.25) |  |
| **Alcohol drinking (%)** |  |  |  | 0.79 |
| Never drinker | 3,954 (58.21) | 2,397 (58.24) | 1,557 (58.16) |  |
| Former drinker | 520 (7.65) | 308 (7.48) | 212 (7.92) |  |
| Current drinker | 2,319 (34.14) | 1,411 (34.28) | 908 (33.92) |  |

**Abbreviations:** BMI, body mass index; CHARLS, China Health and Retirement Longitudinal Study; hs-CRP, high-sensitive C-reactive protein; SD, standard deviation.

**Table S3. Association of baseline hs-CRP and longitudinal hs-CRP changes with incident MetS after multiple imputations of missing data (N= 4,497)**

|  | **Group 1** | **Group 2** | **Group 3** | **Group 4** | **Per 1 log mg/L increase** |
| --- | --- | --- | --- | --- | --- |
| **Hs-CRP quartiles** |  |  |  |  |  |
| Cases/total | 87/1,120 | 141/1,118 | 166/1,131 | 198/1,128 | 592/4,497 |
| OR (95% CI)^a^ | 1.00 (Ref.) | 1.50 (1.12, 2.01) | 1.57 (1.17, 2.09) | 1.83 (1.38, 2.43) | 1.24 (1.11, 1.38) |
| **Groups of absolute changes in hs-CRP during the follow–up** | | |  |  |  |
| Cases/total | 151/1,602 | 90/957 | 151/957 | 200/981 | - |
| OR (95% CI)^a^ | 1.00 (Ref.) | 1.07 (0.80, 1.43) | 2.03 (1.57, 2.63) | 2.66 (2.08, 3.40) | - |
| **Groups of percent changes in hs-CRP during the follow–up** | | |  |  |  |
| Cases/total | 151/1,602 | 130/960 | 135/954 | 176/981 | - |
| OR (95% CI)^a^ | 1.00 (Ref.) | 1.53 (1.17, 1.99) | 1.69 (1.30, 2.20) | 2.56 (1.99, 3.29） | - |

**Abbreviations:** CI, confidence interval; hs-CRP, high-sensitive C-reactive protein; MetS, metabolic syndrome; OR, odds ratio;

^a^ All models were adjusted for age (continuous, years), sex (male and female), education level (illiterate, primary school, middle school, and high school or above), and residence (urban and rural), cigarette smoking (never, former, and current), alcohol drinking (never, former, and current), BMI (continuous, kg/m^2^), hypertension (yes and no), dyslipidemia (yes and no), diabetes (yes and no), heart disease (yes and no) and stroke (yes and no).

**Table S4. Association of baseline hs-CRP and longitudinal hs-CRP changes and incident MetS after excluding participants with diabetes, heart disease and stroke (N= 3,561)**

| **Models** | **Case / total (%)** | **Model 1** | **Model 2** | **Model 3** |
| --- | --- | --- | --- | --- |
|  |  | **OR (95% CI)** | **OR (95% CI)** | **OR (95% CI)** |
| **Hs-CRP group** |  |  |  |  |
| Quartile 1 (< 0.48) | 67/904 (7.41) | 1.00 (Ref.) | 1.00 (Ref.) | 1.00 (Ref.) |
| Quartile 2 (≥ 0.48 to < 0.81) | 107/899 (11.90) | 1.69 (1.23, 2.33) | 1.78 (1.30, 2.46) | 2.56 (1.90, 3.50) |
| Quartile 3 (≥ 0.81 to < 1.63) | 111/889 (12.49) | 1.69 (1.23, 2.34) | 1.80 (1.31, 2.49) | 2.59 (1.91, 3.55) |
| Quartile 4 (≥ 1.63) | 148/869 (17.03) | 1.48 (1.06, 2.08) | 1.45 (1.04, 2.04) | 1.86 (1.35, 2.59) |
| *P* for trend^a^ | 433/3,561 (12.16) | <0.001 | <0.001 | 0.01 |
| **Log (hs-CRP) level** |  |  |  |  |
| Each 1 mg/L increase | 433/3,561 (12.16) | 1.42 (1.27, 1.59) | 1.28 (1.13, 1.45) | 1.25 (1.11, 1.42) |
| **Elevated hs-CRP ^b^** |  |  |  |  |
| No | 308/3,189 (10.09) | 1.00 (Ref.) | 1.00 (Ref.) | 1.00 (Ref.) |
| Yes | 64/372 (17.41) | 1.59 (1.18, 2.11) | 1.43 (1.04, 1.94) | 1.40 (1.01, 1.90) |
| **Groups of absolute changes in hs-CRP during the follow–up^c^** | | |  |  |
| Group1 (≤ 0) | 112/1,279 (8.76) | 1.00 (Ref.) | 1.00 (Ref.) | 1.00 (Ref.) |
| Group 2 (> 0 to ≤ 0.41) | 62/758 (8.18) | 1.36 (0.95, 1.93) | 2.62 (1.92, 3.59) | 3.22 (2.43, 4.30) |
| Group 3 (> 0.41 to ≤ 1.21) | 110/739 (14.88) | 1.36 (0.94, 1.97) | 2.59 (1.87, 3.59) | 3.34 (2.48, 4.52) |
| Group 4 (> 1.21) | 149/785 (18.98) | 1.38 (0.95, 2.00) | 2.68 (1.93, 3.73) | 3.30 (2.44, 4.49) |
| *P* for trend^a^ | 433/3,561 (12.16) | <0.001 | <0.001 | <0.001 |
| **Groups of percent changes in hs-CRP during the follow–up^d^** | |  |  |  |
| Group 1 (≤ 0) | 112/1,279 (8.76) | 1.00 (Ref.) | 1.00 (Ref.) | 1.00 (Ref.) |
| Group 2 (> 0 to ≤ 60%) | 90/737 (12.21) | 1.45 (1.08, 1.94) | 1.57 (1.18, 2.09) | 2.10 (1.60, 2.75) |
| Group 3 (> 60% to ≤ 181%) | 100/764 (13.09) | 1.45 (1.07, 1.98) | 1.60 (1.18, 2.16) | 2.47 (1.86, 3.28) |
| Group 4 (> 181%) | 131/781 (16.77) | 1.50 (1.10, 2.05) | 1.64 (1.21, 2.22) | 2.51 (1.88, 3.36) |
| *P* for trend^a^ | 433/3,561 (12.16) | <0.001 | <0.001 | <0.001 |

**Abbreviations:** CHARLS, China Health and Retirement Longitudinal Study; CI, confidence interval; hs-CRP, high-sensitive C-reactive protein; MetS, metabolic syndrome; OR, odds ratio.

**Model 1:** Non-adjusted.

**Model 2:** Adjusted for age (continuous, years), sex (male and female), education level (illiterate, primary school, middle school, and high school or above), and residence (urban and rural).

**Model 3:** Adjusted for the variables in Model 2 and cigarette smoking (never, former, and current), alcohol drinking (never, former, and current), BMI (continuous, kg/m^2^), hypertension (yes and no), dyslipidemia (yes and no), diabetes (yes and no), heart disease (yes and no) and stroke (yes and no).

^a^ *P* values for trend were estimated by modelling serum hs-CRP using the median for each quartile.

^b^ Elevated hs-CRP was categorized into two groups: Yes (higher than 3 mg/L) and no (less than 3 mg/L).

^c^ Participants were categorized into four groups: Group 1 included participants with negative or null changes, and Group 2-4 were tertiles for the remaining participants.

^d^ Participants were categorized into four groups: Group 1 included participants with negative or null percent changes, and Group 2-4 were tertiles for the remaining participants.

**Table S5. Association between baseline hs-CRP and components of MetS among age, sex and BMI subgroups (N = 4,116)**

| **Subgroups** | **Quartiles of hs-CRP at baseline** | | | | **Per 1 log mg/L increase** | ***P* for interaction^a^** |
| --- | --- | --- | --- | --- | --- | --- |
|  | **Quartile 1** | **Quartile 2** | **Quartile 3** | **Quartile 4** |  |  |
| **Elevated WC** |  |  |  |  |  |  |
| 45-60 years | 1.00 (Ref.) | 0.98 (0.69, 1.38) | 1.29 (0.92, 1.81) | 1.45 (1.03, 2.05) | 1.21 (1.05, 1.39) | 0.69 |
| >60 years | 1.00 (Ref.) | 1.45 (0.90, 2.39) | 1.02 (0.62, 1.70) | 1.31 (0.81, 2.14) | 1.04 (0.86, 1.25) |  |
| Male | 1.00 (Ref.) | 1.23 (0.77, 1.97) | 1.05 (0.66, 1.67) | 1.67 (1.08, 2.61) | 1.18 (0.99, 1.40) | 0.46 |
| Female | 1.00 (Ref.) | 1.05 (0.74, 1.50) | 1.27 (0.90, 1.81) | 1.27 (0.87, 1.83) | 1.15 (0.99, 1.34) |  |
| BMI < 24.0 kg/m^2^ | 1.00 (Ref.) | 1.13 (0.83, 1.56) | 1.32 (0.96, 1.80) | 1.48 (1.08, 2.05) | 1.19 (1.05, 1.35) | 0.88 |
| BMI ≥ 24.0 kg/m^2^ | 1.00 (Ref.) | 1.52 (0.85, 2.72) | 1.51 (0.86, 2.69) | 2.02 (1.15, 3.57) | 1.24 (0.99, 1.57) |  |
| **Elevated TG** |  |  |  |  |  |  |
| 45-60 years | 1.00 (Ref.) | 1.14 (0.87, 1.51) | 1.33 (1.01, 1.75) | 1.34 (1.01, 1.77) | 1.14 (1.02, 1.28) | 0.50 |
| >60 years | 1.00 (Ref.) | 0.97 (0.65, 1.44) | 0.92 (0.63, 1.37) | 1.18 (0.81, 1.73) | 1.10 (0.95, 1.27) |  |
| Male | 1.00 (Ref.) | 1.02 (0.69, 1.49) | 1.17 (0.82, 1.69) | 1.41 (0.99, 2.03) | 1.19 (1.03, 1.37) | 0.80 |
| Female | 1.00 (Ref.) | 1.16 (0.87, 1.54) | 1.19 (0.89, 1.59) | 1.26 (0.93, 1.69) | 1.10 (0.98, 1.24) |  |
| BMI < 24.0 kg/m^2^ | 1.00 (Ref.) | 1.03 (0.77, 1.38) | 1.23 (0.92, 1.64) | 1.48 (1.10, 1.99) | 1.16 (1.04, 1.31) | 0.19 |
| BMI ≥ 24.0 kg/m^2^ | 1.00 (Ref.) | 1.26 (0.87, 1.85) | 1.19 (0.83, 1.73) | 1.31 (0.92, 1.89) | 1.15 (1.00, 1.32) |  |
| **Reduced HDL-C** |  |  |  |  |  |  |
| 45-60 years | 1.00 (Ref.) | 1.51 (0.97, 2.36) | 1.11 (0.69, 1.78) | 1.07 (0.65, 1.74) | 1.02 (0.84, 1.24) | 0.84 |
| >60 years | 1.00 (Ref.) | 0.89 (0.52, 1.51) | 0.87 (0.52, 1.46) | 0.75 (0.45, 1.26) | 0.90 (0.73, 1.10) |  |
| Male | 1.00 (Ref.) | 1.05 (0.67, 1.68) | 1.01 (0.64, 1.61) | 0.97 (0.62, 1.55) | 0.98 (0.81, 1.17) | 0.66 |
| Female | 1.00 (Ref.) | 1.31 (0.80, 2.17) | 0.95 (0.56, 1.62) | 0.83 (0.47, 1.45) | 0.96 (0.77, 1.19) |  |
| BMI < 24.0 kg/m^2^ | 1.00 (Ref.) | 0.96 (0.63, 1.46) | 0.99 (0.64, 1.50) | 1.08 (0.70, 1.65) | 1.07 (0.90, 1.27) | 0.21 |
| BMI ≥ 24.0 kg/m^2^ | 1.00 (Ref.) | 1.75 (0.96, 3.37) | 1.13 (0.60, 2.20) | 0.88 (0.46, 1.72) | 0.83 (0.65, 1.05) |  |
| **Elevated BP** |  |  |  |  |  |  |
| 45-60 years | 1.00 (Ref.) | 1.18 (0.87, 1.60) | 0.96 (0.70, 1.31) | 0.92 (0.67, 1.27) | 0.97 (0.85, 1.11) | 0.73 |
| >60 years | 1.00 (Ref.) | 1.08 (0.70, 1.69) | 1.09 (0.71, 1.67) | 1.38 (0.91, 2.10) | 1.18 (1.00, 1.39) |  |
| Male | 1.00 (Ref.) | 1.20 (0.83, 1.74) | 0.88 (0.61, 1.27) | 0.91 (0.63, 1.30) | 0.97 (0.84, 1.12) | 0.24 |
| Female | 1.00 (Ref.) | 1.06 (0.75, 1.50) | 1.06 (0.75, 1.51) | 1.19 (0.83, 1.70) | 1.09 (0.94, 1.26) |  |
| BMI < 24.0 kg/m^2^ | 1.00 (Ref.) | 1.08 (0.81, 1.45) | 0.87 (0.64, 1.18) | 1.00 (0.74, 1.35) | 1.03 (0.91, 1.16) | 0.66 |
| BMI ≥ 24.0 kg/m^2^ | 1.00 (Ref.) | 1.50 (0.91, 2.49) | 1.56 (0.96, 2.57) | 1.44 (0.89, 2.35) | 1.10 (0.92, 1.33) |  |
| **Elevated FBG** |  |  |  |  |  |  |
| 45-60 years | 1.00 (Ref.) | 1.29 (0.87, 1.90) | 1.59 (1.08, 2.34) | 1.65 (1.12, 2.44) | 1.26 (1.08, 1.46) | 0.34 |
| >60 years | 1.00 (Ref.) | 1.09 (0.68, 1.76) | 1.21 (0.78, 1.92) | 1.50 (0.97, 2.36) | 1.20 (1.02, 1.41) |  |
| Male | 1.00 (Ref.) | 1.07 (0.69, 1.66) | 1.29 (0.85, 1.97) | 1.32 (0.88, 2.02) | 1.14 (0.97, 1.34) | 0.50 |
| Female | 1.00 (Ref.) | 1.33 (0.87, 2.02) | 1.52 (1.02, 2.30) | 1.81 (1.21, 2.74) | 1.28 (1.10, 1.50) |  |
| BMI < 24.0 kg/m^2^ | 1.00 (Ref.) | 1.19 (0.83, 1.72) | 1.25 (0.86, 1.81) | 1.69 (1.18, 2.43) | 1.22 (1.06, 1.40) | 0.86 |
| BMI ≥ 24.0 kg/m^2^ | 1.00 (Ref.) | 1.27 (0.75, 2.20) | 1.69 (1.03, 2.84) | 1.56 (0.96, 2.63) | 1.23 (1.03, 1.47) |  |

**Abbreviations:** BMI, body mass index; BP, blood pressure; CHARLS, China Health and Retirement Longitudinal Study; FBG, fasting blood glucose; HDL-C, high-density lipoprotein cholesterol; hs-CRP, high-sensitive C-reactive protein; MetS, metabolic syndrome; TG, triglycerides; WC, waist circumference.

^a^ *P* values for interaction were estimated using the likelihood ratio test for the product term of the stratifying variable and hs-CRP quartiles to the main model.

All models were adjusted for age (continuous, years), sex (male and female), education level (illiterate, primary school, middle school, and high school or above), and residence (urban and rural), cigarette smoking (never, former, and current), alcohol drinking (never, former, and current), BMI (continuous, kg/m^2^), hypertension (yes and no), dyslipidemia (yes and no), diabetes (yes and no), heart disease (yes and no) and stroke (yes and no).

**Table S6. Association between longitudinal hs-CRP changes and components of MetS among age, sex and BMI subgroups**

| **Subgroups** | **Groups of absolute changes in hs-CRP during the follow–up** | | | | ***P* for interaction^a^** |
| --- | --- | --- | --- | --- | --- |
|  | **Group 1** | **Group 2** | **Group 3** | **Group 4** |  |
| **Elevated WC** |  |  |  |  |  |
| 45-60 years | 1.00 (Ref.) | 1.02 (0.73, 1.44) | 1.89 (1.37, 2.61) | 1.36 (0.97, 1.90) | 0.46 |
| >60 years | 1.00 (Ref.) | 1.32 (0.81, 2.14) | 1.88 (1.21, 2.91) | 2.51 (1.63, 3.88) |  |
| Male | 1.00 (Ref.) | 0.68 (0.42, 1.08) | 1.62 (1.09, 2.41) | 1.69 (1.14, 2.48) | 0.04 |
| Female | 1.00 (Ref.) | 1.51 (1.06, 2.15) | 2.03 (1.45, 2.86) | 1.74 (1.22, 2.49) |  |
| BMI < 24.0 kg/m^2^ | 1.00 (Ref.) | 1.38 (1.00, 1.91) | 2.00 (1.48, 2.72) | 1.92 (1.42, 2.61) | <0.001 |
| BMI ≥ 24.0 kg/m^2^ | 1.00 (Ref.) | 0.67 (0.40, 1.13) | 1.72 (1.05, 2.84) | 1.39 (0.82, 2.36) |  |
| **Elevated TG** |  |  |  |  |  |
| 45-60 years | 1.00 (Ref.) | 1.27 (0.94, 1.71) | 2.54 (1.93, 3.33) | 3.71 (2.84, 4.87) | 0.01 |
| >60 years | 1.00 (Ref.) | 1.09 (0.72, 1.62) | 2.67 (1.89, 3.77) | 2.33 (1.66, 3.28) |  |
| Male | 1.00 (Ref.) | 1.21 (0.83, 1.76) | 2.21 (1.57, 3.12) | 3.01 (2.18, 4.17) | 0.72 |
| Female | 1.00 (Ref.) | 1.18 (0.86, 1.61) | 2.82 (2.15, 3.72) | 3.21 (2.44, 4.23) |  |
| BMI < 24.0 kg/m^2^ | 1.00 (Ref.) | 1.16 (0.84, 1.61) | 2.82 (2.12, 3.76) | 3.19 (2.40, 4.26) | 0.55 |
| BMI ≥ 24.0 kg/m^2^ | 1.00 (Ref.) | 1.23 (0.86, 1.76) | 2.22 (1.62, 3.06) | 2.87 (2.11, 3.91) |  |
| **Reduced HDL-C** |  |  |  |  |  |
| 45-60 years | 1.00 (Ref.) | 0.91 (0.51, 1.56) | 1.57 (0.97, 2.55) | 3.49 (2.31, 5.37) | 0.12 |
| >60 years | 1.00 (Ref.) | 2.18 (1.29, 3.69) | 1.57 (0.89, 2.74) | 3.50 (2.21, 5.66) |  |
| Male | 1.00 (Ref.) | 1.31 (0.79, 2.15) | 1.65 (1.01, 2.67) | 3.76 (2.50, 5.73) | 0.90 |
| Female | 1.00 (Ref.) | 1.44 (0.81, 2.53) | 1.46 (0.83, 2.56) | 3.35 (2.10, 5.47) |  |
| BMI < 24.0 kg/m^2^ | 1.00 (Ref.) | 1.55 (0.97, 2.48) | 1.64 (1.01, 2.64) | 3.52 (2.35, 5.34) | 0.47 |
| BMI ≥ 24.0 kg/m^2^ | 1.00 (Ref.) | 1.15 (0.59, 2.15) | 1.50 (0.85, 2.63) | 3.45 (2.14, 5.66) |  |
| **Elevated BP** |  |  |  |  |  |
| 45-60 years | 1.00 (Ref.) | 1.09 (0.80, 1.49) | 1.42 (1.05, 1.91) | 1.36 (1.01, 1.84) | 0.02 |
| >60 years | 1.00 (Ref.) | 1.25 (0.85, 1.83) | 0.68 (0.45, 1.01) | 0.87 (0.59, 1.27) |  |
| Male | 1.00 (Ref.) | 1.17 (0.83, 1.64) | 1.25 (0.88, 1.77) | 1.03 (0.74, 1.45) | 0.35 |
| Female | 1.00 (Ref.) | 1.10 (0.79, 1.54) | 1.04 (0.74, 1.45) | 1.31 (0.94, 1.82) |  |
| BMI < 24.0 kg/m^2^ | 1.00 (Ref.) | 1.21 (0.91, 1.61) | 1.20 (0.90, 1.61) | 1.04 (0.77, 1.39) | 0.83 |
| BMI ≥ 24.0 kg/m^2^ | 1.00 (Ref.) | 0.94 (0.61, 1.45) | 0.96 (0.63, 1.46) | 1.29 (0.87, 1.91) |  |
| **Elevated FBG** |  |  |  |  |  |
| 45-60 years | 1.00 (Ref.) | 0.64 (0.42, 0.95) | 1.00 (0.69, 1.43) | 1.66 (1.20, 2.30) | 0.41 |
| >60 years | 1.00 (Ref.) | 0.67 (0.42, 1.05) | 1.38 (0.93, 2.03) | 1.47 (1.02, 2.13) |  |
| Male | 1.00 (Ref.) | 0.71 (0.45, 1.10) | 1.55 (1.06, 2.25) | 1.55 (1.08, 2.22) | 0.12 |
| Female | 1.00 (Ref.) | 0.61 (0.40, 0.93) | 0.88 (0.60, 1.28) | 1.62 (1.16, 2.26) |  |
| BMI < 24.0 kg/m^2^ | 1.00 (Ref.) | 0.52 (0.34, 0.78) | 1.24 (0.88, 1.74) | 1.43 (1.03, 1.98) | 0.09 |
| BMI ≥ 24.0 kg/m^2^ | 1.00 (Ref.) | 0.91 (0.57, 1.43) | 1.09 (0.72, 1.65) | 1.90 (1.31, 2.76) |  |

**Abbreviations:** BMI, body mass index; BP, blood pressure; FBG, fasting blood glucose; HDL-C, high-density lipoprotein cholesterol; hs-CRP, high-sensitive C-reactive protein; MetS, metabolic syndrome; TG, triglycerides; WC, waist circumference.

^a^ *P* values for interaction were estimated using the likelihood ratio test for the product term of the stratifying variable and hs-CRP change groups to the main model.

All models were adjusted for age (continuous, years), sex (male and female), education level (illiterate, primary school, middle school, and high school or above), and residence (urban and rural), cigarette smoking (never, former, and current), alcohol drinking (never, former, and current), BMI (continuous, kg/m^2^), hypertension (yes and no), dyslipidemia (yes and no), diabetes (yes and no), heart disease (yes and no) and stroke (yes and no).

**Table S7. Basic information of studies included in the meta-analysis**

| Study | Country/  region | Age range | Cases/  sample | Duration (years) | MetS definition | Reference | Effect type | Effect size | Covariates adjusted |
| --- | --- | --- | --- | --- | --- | --- | --- | --- | --- |
| Xue (2021) | China | ≥45 y | 535/4,116 | 4 | Chinese | Quartile 4 vs. 1 | HR | 1.83 (1.37, 2.47) | age, sex, marital status, education, and residence, cigarette smoking, alcohol drinking, BMI, diabetes, hypertension, dyslipidemia, heart disease, and stroke |
|  |  |  |  |  |  | Per 1 log mg/L | HR | 1.23 (1.10-1.38) |  |
| Hong (2020) [1] | China | >18 y | 116/886 | 5 | Chinese | Quartile 4 vs. 1 | OR | All: 4.06 (1.91-8.65)^a^ | age, sex, education, exercise, smoking, alcohol consumption, and eGFR |
|  |  |  |  |  |  |  |  | Men: 3.15 (0.82–12.10) |  |
|  |  |  |  |  |  |  |  | Women: 4.82 (1.89–12.3) |  |
| Yoon (2018)[2] | Korea | NA | 134/3,386 | 7 | NCEP-ATP III | Tertile 3 vs. 1 | HR | Men: 1.47 (0.96-2.24) | age, smoking status, alcohol intake, and exercise |
| Saisho (2013)[3] | Japan | ≥40 y | 61/750 | 4 | NCEP-ATP III | Per 1 log mg/L | OR | 1.25 (0.94-1.67) | age, sex, waist circumference, smoking status, alcohol intake, exercise, γ-GTP, LDL-C, Uric acid, and HMW-adiponectin |
|  |  |  |  |  | Japanese | Per 1 log mg/L | OR | 1.29 (0.94-1.78) |  |
| Oda (2013)[4] | Japan | NA | 224/2,383 | 4 | NCEP-ATP III | Per 1 log mg/L | HR | Men: 1.00 (0.84-1.20) | age, smoking, drinking, physical activity, use of antihypertensive, antihyperlipidemic medications, abdominal obesity, blood pressure, fasting glucose, triglycerides, and HDL-C |
|  |  |  |  |  |  | Per 1 log mg/L | HR | Women: 0.96 (0.73-1.25) |  |
| Musani (2013)[5] | US | 21-95 y | 278/1,215 | 11 | NCEP-ATP III | Per 1 log mg/L | OR | 1.21 (1.02-1.43) | age, sex, SBP, diastolic BP, waist circumference, HDL-C, fasting triglycerides, fasting plasma glucose, and sex hormone use |
|  |  |  |  |  |  | Quartile 4 vs. Quartile 2-3 | OR | 1.91 (1.31–2.80) |  |
| Bo (2009)[6] | Italy | 45–64 y | 28/201 | 4.5 | NCEP-ATPIII | Per 1 log mg/L | OR | 4.05 (2.23-7.38) | sex, smoking, education and exercise level |
| Lim (2005)[7] | Korea | 40-65 y | 2,540/9,773 | NA | NCEP-ATP III | highest vs. lowest | OR | 1.72 (1.47-2.02) | age, sex, BMI and smoking status |
| Laaksonen (2004)[8] | Finland | 42, 48, 54, 60 y | 103/598 | 11 | NCEP-ATP III | Tertile 3 vs. 1 | OR | Men: 1.36 (0.72-2.57) | age, cardiovascular disease, socioeconomic status, physical activity, alcohol consumption, smoking, BMI, waist girth, levels of insulin, glucose and triglycerides, systolic blood pressure, and blood pressure medication |
|  |  |  |  |  | WHO | Tertile 3 vs. 1 | OR | Men: 1.85 (0.96-3.58) |  |
| Han (2002)[9] | Mexico | 35-64 y | 190/1,244 | 6 | WHO | Tertile 3 vs. 1 | OR | Men: 0.9 (0.5-2.0) | age, cigarette smoking, alcohol consumption, and physical activity |
|  |  |  |  |  |  | Tertile 3 vs. 1 | OR | Women: 4.1 (2.1-8.0) |  |

**Abbreviations**: BMI, body mass index; BP, blood pressure; eGFR, epidermal growth factor receptor; γ-GTP, gamma-glutamyl transpeptidase; HR, hazard ratio; HDL-C, high-density lipoprotein cholesterol; IDF-MetS, International Diabetes Federation (IDF)- metabolic syndrome; LDL-C, low-density lipoprotein cholesterol; MetS, metabolic syndrome; NCEP ATP III, National Cholesterol Education Program Adult Treatment Panel III criteria; OR, odds ratio; WHO, World Health Organization.

**Table S8. Quality assessment for the 11 included studies in the meta-analysis**

| Author  (year) | Selection | | | | Comparability | | Outcome | | | Total |
| --- | --- | --- | --- | --- | --- | --- | --- | --- | --- | --- |
|  | Representativeness of the exposed cohort | Selection of the non-exposed cohort | Ascertainment of exposure | Outcome of interest not present at start of the study | Control for primary confounders | Control for secondary confounders | Assessment of outcome | Duration of follow-up | Adequacy of follow-up |  |
| Xue (2021) | 1 | 1 | 1 | 1 | 0 | 1 | 1 | 0 | 0 | 6 |
| Hong (2020) | 1 | 1 | 1 | 1 | 1 | 0 | 1 | 1 | 0 | 8 |
| Yoon (2018) | 0 | 1 | 1 | 1 | 1 | 0 | 1 | 1 | 1 | 8 |
| Saisho (2013) | 0 | 1 | 1 | 1 | 0 | 0 | 1 | 0 | 0 | 5 |
| Oda (2013) | 0 | 1 | 1 | 1 | 1 | 1 | 1 | 0 | 0 | 6 |
| Musani (2013) | 1 | 1 | 1 | 1 | 1 | 1 | 1 | 1 | 0 | 8 |
| Bo (2009） | 0 | 1 | 1 | 1 | 0 | 0 | 1 | 0 | 1 | 5 |
| Lim (2005) | 1 | 1 | 1 | 1 | 0 | 0 | 1 | 0 | 1 | 6 |
| Laaksonen (2004) | 0 | 1 | 1 | 1 | 1 | 1 | 1 | 1 | 0 | 7 |
| Han (2002） | 1 | 1 | 1 | 1 | 1 | 0 | 1 | 1 | 0 | 7 |

* Representativeness of the exposed cohort: 1 point awarded if community-based population; Selection of the non-exposed cohort: 1 point awarded if drawn from the same community as the exposed cohort; Ascertainment of exposure: 1 point awarded if hs-CRP at baseline was identified by researchers; Outcome of interest not present at start of the study: 1 point awarded if individuals with prevalent MetS at baseline were excluded; Control for primary confounders: 1 point awarded if adjustment for age, sex, BMI, smoking status, alcohol intake, and physical activity; Control for secondary confounders: 1 point awarded if adjustment for further disease-related confounders; Assessment of outcome: 1 point awarded if MetS was identified by standard criteria; Duration of follow-up; 1 point awarded if follow-up ≥ 5 years; Adequacy of follow-up: 1 point awarded if loss to follow-up <20%.

The China Health and Retirement Longitudinal Study

(N=17,708, 2011-2012)

Included for analyses (n=4,116)

Loss to follow up in 2015-2016 (n=2,219)

Availability of questionnaires and laboratory measurements at baseline (n=11,847)

Lack of information of MetS at baseline (n=2,028)

With high-sensitive CRP >10 mg/L at baseline (n=276)

With MetS at baseline (n=2,740)

Lack of baseline information of major behaviors or lifestyle covariates (n=381), including age (n=177), residential area (n=4), cigarette smoking (n=12), alcohol drinking (n=3), BMI (n=33), diabetes (n=60), hypertension (n=19), dyslipidemia (n=53), heart diseases (n=12), stroke (n=8).

Lack of information of MetS or hs-CRP in 2015-2016 (n=87)

Eligible participants (n=6,803)

**Figure S1. Flowchart of inclusion and exclusion of study participants**

**Abbreviations:** BMI, body mass index; hs-CRP, high-sensitive C-reactive protein; MetS, metabolic syndrome.

Number of records identified by database searching through PubMed and Embase: 9,902

**Screening**

**Included**

**Eligibility**

**Identification**

Number of duplicate records removed: 2,514

Number of records screened: 7,388

Number of articles excluded based on the title and abstract: 7,324

Number of full-text articles examined: 64

55 full-text articles excluded:

1. Non-cohort studies (n=49)
2. Reviews (n=1)
3. Conference abstracts (n=2)
4. Letters to editor (n=2)
5. Not report the effect estimate (n =1)

10 studies included in the meta-analysis (including our cohort study)

**Figure S2. Flowchart of literature identification for the meta-analysis**

**Figure S3.** **Association of combined baseline hs-CRP and longitudinal hs-CRP changes with incident MetS**

**Abbreviations:** CI, confidence interval; OR, odd ratio; hs-CRP, high-density C-reactive protein; MetS, metabolic syndrome.

Adjusted for age (continuous, years), sex (male and female), education level (illiterate, primary school, middle school, and high school or above), and residence (urban and rural), cigarette smoking (never, former, and current), alcohol drinking (never, former, and current), BMI (continuous, kg/m^2^), hypertension (yes and no), dyslipidemia (yes and no), diabetes (yes and no), heart disease (yes and no) and stroke (yes and no).


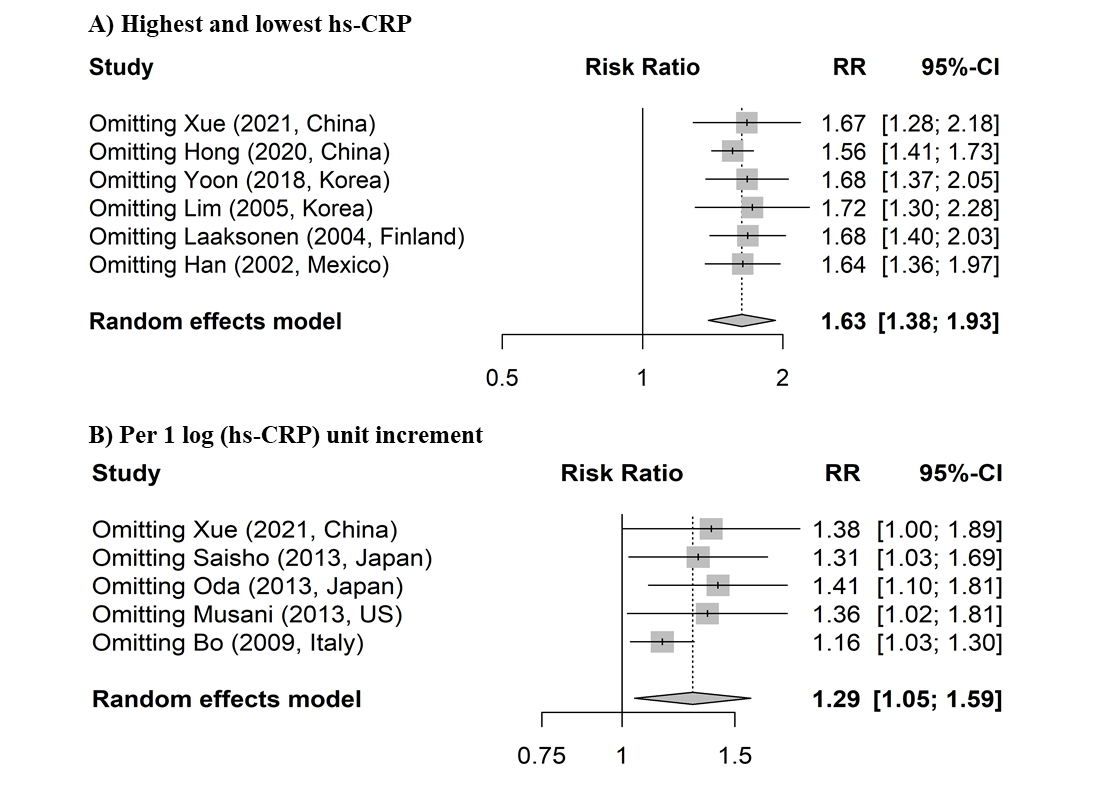


Figure S4. **Sensitivity analyses for the association between hs-CRP and incident MetS with exclusions of each study a time.** A) Comparing the highest and lowest serum hs-CRP; and B) Using log-transformed serum hs-CRP. **Abbreviations:** CI, confidence interval; hs-CRP, high-sensitive C-reactive protein; MetS, metabolic syndrome; RR, relative ratio.


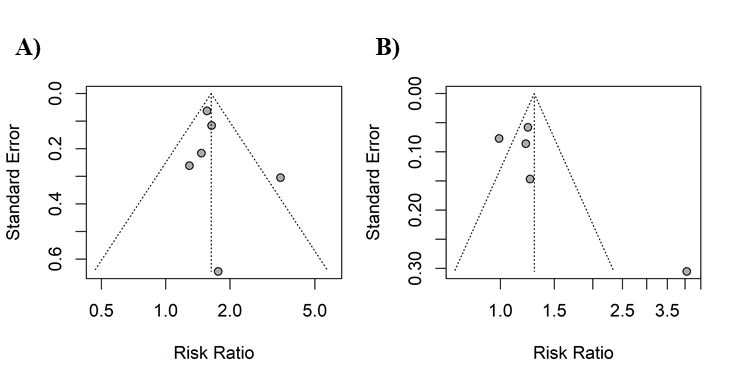


**Figure S5.** **Funnel plot for assessment of publication bias for the association between hs-CRP and MetS.** A) Comparing the highest and lowest serum hs-CRP (*P* value for Egger’s test = 0.51); and B) Using log-transformed serum hs-CRP (*P* value for Egger’s test = 0.22).

**Abbreviations:** hs-CRP, high-sensitivity C-reactive protein; MetS, metabolic syndrome.

**Reference**

1. Hong, G.B., et al., *High-sensitivity c-reactive protein leads to increased incident metabolic syndrome in women but not in men: A five-year follow-up study in a Chinese population.* Diabetes, Metabolic Syndrome and Obesity: Targets and Therapy, 2020. **13**: p. 581-590.

2. Yoon, K., et al., *Higher and increased concentration of hs-CRP within normal range can predict the incidence of metabolic syndrome in healthy men.* Diabetes and Metabolic Syndrome: Clinical Research and Reviews, 2018. **12**(6): p. 977-983.

3. Saisho, Y., et al., *C-Reactive Protein, High-Molecular-Weight Adiponectin and Development of Metabolic Syndrome in the Japanese General Population: A Longitudinal Cohort Study.* PLoS ONE, 2013. **8**(9): p. e73430.

4. Oda, E., *High-sensitivity C-reactive protein and white blood cell count equally predict development of the metabolic syndrome in a Japanese health screening population.* Acta Diabetologica, 2013. **50**(4): p. 633-638.

5. Musani, S.K., et al., *Aldosterone, c-reactive protein, and plasma b-type natriuretic peptide are associated with the development of metabolic syndrome and longitudinal changes in metabolic syndrome components : Findings from the jackson heart study.* Diabetes Care, 2013. **36**(10): p. 3084-3092.

6. Bo, S., et al., *What predicts the occurrence of the metabolic syndrome in a population-based cohort of adult healthy subjects?* Diabetes/Metabolism Research and Reviews, 2009. **25**(1): p. 76-82.

7. Lim, S., et al., *C-reactive protein level as an independent risk factor of metabolic syndrome in the Korean population CRP as risk factor of metabolic syndrome.* Diabetes Research and Clinical Practice, 2005. **70**(2): p. 126-133.

8. Laaksonen, D.E., et al., *C-reactive protein and the development of the metabolic syndrome and diabetes in middle-aged men.* Diabetologia, 2004. **47**(8): p. 1403-10.

9. Han, T.S., et al., *Prospective study of C-reactive protein in relation to the development of diabetes and metabolic syndrome in the Mexico City diabetes study.* Diabetes Care, 2002. **25**(11): p. 2016-2021.
